# A quality improvement project to improve the physical health of people with intellectual disability & severe mental illness in a forensic inpatient ward

**DOI:** 10.1192/bjo.2021.495

**Published:** 2021-06-18

**Authors:** Rupal Dave, Rachael Miller, Lucy Bermange, Laura Humphries

**Affiliations:** 1 East London NHS Foundation Trust; 2 John Howard Centre for Forensic Mental Health

## Abstract

**Aims:**

Review physical health risk factors of service users Co-produce personalised care plans for service users Improve health knowledge and confidence in self-management of health problems Support reduction in risk by improving physical activity levels and supporting healthy dietary choices.

**Background:**

People with intellectual disability have poorer physical health outcomes than those without intellectual disability; there is higher prevalence of obesity, constipation and diabetes in this group of the population, and consistent evidence of premature mortality. Excess mortality in persons with severe mental illness has also been established.

Empowering patients to take an active role in their care, is good practice and encouraged as part of the NHS Long Term Plan.

Quality Improvement methodology was used to design and deliver a multi-disciplinary team (MDT) intervention, on a forensic mental health ward for persons with intellectual disability, to improve physical health in this patient group.

**Method:**

Cardiovascular risk was assessed for 13 patients on a low secure forensic mental health ward. Measures of weight, BMI, blood pressure, resting heart rate, smoking status & status regarding prescription of psychotropic medications were collected.

Together with individual comorbidities and activity levels, a personalised care plan was co-produced by MDT members and patients. Motivational interviewing techniques were adapted to support patients to set personal goals.

Education sessions were designed in 'easy-read' format and delivered by MDT members in a group format. Focus groups were held with service users and with staff members to explore barriers to change. Based on these, specific ideas to increase physical activity and support healthy dietary changes were introduced.

The Patient Activation Measure (PAM) questionnaire was modified and used to assess confidence and knowledge in preventing or reducing health problems, and maintaining changes.

**Result:**

Cardiovascular risk and activity levels were assessed for 13 inpatients. 85% of patients had a BMI in the overweight or obese range. 62% were regular cigarette smokers. 92% were prescribed psychotropic medications. On review of 2 months of opportunities for activity, all patients were categorised as 'inactive'. Patients engaged to varying degrees to co-produce personalised care plans and to engage in group education and physical activity. Of these patients, all showed improvement in measures of Patient Activation and activity level.

**Conclusion:**

An individualised approach is required in exploring physical health problems, considering modifiable risk factors and addressing barriers to change. Co-production, and active participation of MDT members in role-modelling 'healthy habits' was positively reported by patients to facilitate self-management.

